# Chemical priming potentiates mesothelin-targeting chimeric antigen receptor-engineered NK-92 antitumor activity by improving tumor trafficking and cytotoxic killing dynamics

**DOI:** 10.3389/fimmu.2026.1860442

**Published:** 2026-06-01

**Authors:** Ki Seo Ryu, Hail Park, Eunchong Maeng, Jun-Yeon Lim, Duck Cho, Seung Hee Choi, Kyung-Soon Park

**Affiliations:** 1 Department of Biomedical Science, Division of Life Science, CHA University, Seongnam-si, Republic of Korea; 2 Department of Laboratory Medicine and Genetics, Samsung Medical Center, School of Medicine, Sungkyunkwan University, Seoul, Republic of Korea; 3 Northwestern Medicine Malnati Brain Tumor Institute of the Lurie Comprehensive Cancer Center, Feinberg School of Medicine, Northwestern University, Chicago, IL, United States; 4 Department of Neurological Surgery, Northwestern University, Feinberg School of Medicine, Chicago, IL, United States

**Keywords:** 25 kDa branched polyethylenimine, chemical priming, chimeric antigen receptor-engineered natural killer cells, mesothelin, ovarian cancer, solid tumor

## Abstract

**Introduction:**

Chimeric antigen receptor-engineered natural killer (CAR-NK) cells have emerged as a promising strategy for cancer immunotherapy; however, their efficacy against solid tumors remains limited by inefficient tumor trafficking and impaired cytotoxic function within the tumor microenvironment. Here, we investigated whether a non-genetic chemical priming strategy could pre-arm CAR-NK cells and enhance their migratory and cytotoxic functions.

**Methods:**

Based on our previous findings that transient exposure to 25 kDa branched polyethylenimine (25KbPEI) induces a primed phenotype, mesothelin-targeting CAR-NK-92 cells were chemically primed and evaluated for migration, cytotoxicity, killing dynamics, perforin accumulation, cytokine production, and in vivo antitumor efficacy in a SKOV3 xenograft model.

**Results:**

Chemical priming significantly enhanced cytotoxicity and degranulation against ovarian cancer cells without compromising cell viability. Primed CAR-NK cells showed increased CCR7 expression and improved tumor-directed migration. Live-cell imaging further revealed accelerated target engagement and shortened killing time, indicating enhanced cytotoxic kinetics. In addition, chemical priming increased perforin accumulation and IFN-γ production. In the SKOV3 xenograft model, primed CAR-NK cells achieved superior tumor control and increased intratumoral infiltration compared with non-primed CAR-NK cells, while maintaining a favorable safety profile.

**Discussion:**

Collectively, these findings demonstrate that chemical priming enhances CAR-NK cell function and provides a promising non-genetic strategy to improve CAR-NK cell activity against solid tumor models.

## Introduction

Natural killer (NK) cells are cytotoxic innate lymphocytes that eliminate transformed or virally infected cells without prior antigen sensitization (1–3). Unlike T cells, NK cells recognize targets in a major histocompatibility complex (MHC)-independent manner through a dynamic balance of activating and inhibitory receptors (4, 5). Upon activation, NK cells form an immunological synapse and rapidly release lytic granules containing perforin and granzymes to induce apoptosis of target cells (6). In addition to direct cytotoxicity, NK cells secrete cytokines such as interferon-γ (IFN-γ), thereby shaping adaptive immune responses and contributing to tumor immune surveillance (1, 7). These intrinsic properties position NK cells as attractive candidates for adoptive cellular immunotherapy.

Chimeric antigen receptor-engineered NK (CAR-NK) cells have emerged as a promising next-generation platform that combines innate cytotoxicity with antigen specificity (8, 9). Early-phase clinical studies targeting CD19-positive hematologic malignancies have demonstrated encouraging efficacy with a favorable safety profile, including a low incidence of cytokine release syndrome and neurotoxicity (10). In addition, the feasibility of allogeneic ‘off-the-shelf’ manufacturing provides a practical rationale for continued investigation of CAR-NK cell therapy (8, 9). However, extending these successes to solid tumors remains challenging and requires not only appropriate tumor-associated antigens but also strategies to overcome the hostile tumor microenvironment (TME) (11–13).

Despite advances in CAR design and antigen targeting, the efficacy of CAR-NK cells in solid tumors remains suboptimal (11). Antigen recognition alone is insufficient to overcome the multifaceted barriers imposed by the TME. Solid tumors limit NK cell infiltration through abnormal vasculature and dense stromal architecture, while immunosuppressive mediators suppress activation, degranulation, and IFN-γ production (14, 15). In parallel, metabolic stressors, including hypoxia, nutrient deprivation, and acidosis, compromise mitochondrial fitness and reduce effector durability (16, 17). These suppressive factors converge on intrinsic, post-recognition determinants of NK cell function, including efficient trafficking, immunological synapse formation, cytotoxic payload mobilization, and sustained killing capacity (6, 18). Thus, strategies that enhance antigen specificity without reinforcing intrinsic functional readiness are unlikely to achieve robust antitumor efficacy in solid tumors.

Cytokine-based priming approaches, such as IL-15 or IL-12/15/18 stimulation, have been widely explored to enhance NK cell persistence and effector function (19, 20). While these strategies promote transcriptional reprogramming and generate ‘memory-like’ NK cells, they do not adequately address the critical bottlenecks of tumor infiltration and rapid cytotoxic execution within the TME. Notably, cytokine priming primarily enhances signaling competence and survival, whereas immediate effector readiness and trafficking capacity remain limiting. Therefore, alternative strategies that directly reinforce metabolic fitness and cytotoxic readiness prior to tumor encounter are needed.

We previously demonstrated that transient exposure of NK cells to 25 kDa branched polyethylenimine (25KbPEI) induces a chemically primed phenotype characterized by rapid Ca²^+^ influx and enhanced mitochondrial oxidative phosphorylation (OXPHOS), thereby establishing a metabolically reinforced effector state (21, 22). In the present study, we define chemical priming as a short-term, non-genetic preconditioning process in which NK cells are transiently exposed to 25KbPEI prior to target cell encounter. We further define metabolic reinforcement as the accompanying enhancement of mitochondrial OXPHOS that may support NK cell effector functions under stress conditions. Chemically primed NK cells exhibit increased expression of chemokine receptors and enhanced migratory capacity, supporting improved trafficking competence (21). In addition, these cells show increased intracellular perforin accumulation and elevated IFN-γ production, indicating reinforcement of both cytotoxic payload and immunomodulatory function (21, 23, 24). Beyond NK cells, PEI-based or polycation-mediated modulation has also been reported to influence innate immune cell functions in a context-dependent manner, including effects on dendritic cells and macrophages (25, 26). Based on this framework, we hypothesized that chemical priming could functionally pre-arm CAR-NK cells, enabling enhanced tumor trafficking, sustained metabolic fitness, and accelerated cytotoxic execution upon antigen engagement. To test this hypothesis, we generated chemically primed CAR-NK cells (CAR-Chem_NK-92) and performed a comprehensive evaluation of their phenotypic, functional, and therapeutic properties in a solid tumor model. Mesothelin (MSLN), a glycosylphosphatidylinositol-anchored surface protein, is highly overexpressed in ovarian cancer and several other solid malignancies while showing relatively restricted expression in normal tissues (27), making it an attractive target for CAR-based immunotherapy. Previous studies using MSLN-targeted CAR-NK-92 and memory-like CAR-NK platforms have demonstrated antigen-specific antitumor activity in ovarian cancer models (28, 29), supporting the use of MSLN-positive ovarian cancer as a clinically relevant model to evaluate whether chemical priming can further enhance CAR-NK trafficking and cytotoxic function.

Our results demonstrate that chemical priming significantly enhances CAR-mediated cytotoxicity *in vitro* and improves antitumor activity *in vivo*. These findings support chemical priming as a practical and modular strategy to overcome intrinsic functional limitations of CAR-NK cell therapy in solid tumors.

## Materials and methods

### Cell culture

NK-92 cells and mesothelin (MSLN)-CAR–expressing NK-92 cells (MSLN-CAR-NK-92) were used in this study. MSLN-CAR-NK-92 cells were generated by lentiviral transduction of NK-92 cells with an MSLN-specific CAR construct, as described below. The human myelogenous leukemia cell line K562 and the human ovarian cancer cell lines OVCAR3 and SKOV3 were obtained from the American Type Culture Collection (ATCC).

NK-92 and MSLN-CAR-NK-92 cells were cultured in Alpha Minimum Essential Medium (α-MEM; Gibco/Life Technologies, Grand Island, New York) supplemented with 12.5% fetal bovine serum (FBS; Gibco/Life Technologies), 12.5% horse serum (Gibco/Life Technologies), 1% penicillin/streptomycin (Gibco/Life Technologies), 0.2 mM myo-inositol (Sigma-Aldrich, St. Louis, USA), 0.02 mM folic acid (Sigma-Aldrich), 0.1 mM 2-mercaptoethanol (Gibco/Life Technologies), and 200 U/mL IL-2 (PeproTech). K562 cells were cultured in Roswell Park Memorial Institute 1640 (RPMI 1640; Gibco/Life Technologies) supplemented with 10% FBS and 1% penicillin/streptomycin. OVCAR3 cells were cultured in RPMI 1640 medium supplemented with 20% FBS and 1% penicillin/streptomycin. SKOV3 cells were cultured in McCoy’s 5A (modified) medium (SolBio) supplemented with 10% FBS and 1% penicillin/streptomycin. All cells were cultured at 37 °C in a humidified incubator containing 5% CO_2_.

### Generation of MSLN-CAR-NK-92

The MSLN-CAR-NK-92 cells used in the present study were previously established and characterized according to our published protocol (30). The previously established MSLN-targeting CAR construct consisted of a CD8 leader sequence, an SS1-derived anti-MSLN scFv, a CD8 hinge/transmembrane region, and intracellular 4-1BB and CD3ζ signaling domains. In the original study, the SS1 scFv sequence was designed based on the publicly available SS1 sequence (US10640569), synthesized with XbaI and EcoRI restriction sites, and subsequently cloned into a pCDH-MSCV backbone vector (System Biosciences, Palo Alto, CA, USA).

### Generation of CAR-Chem_NK-92

CAR-Chem_NK-92 cells were generated by treating MSLN-CAR-NK-92 cells with 25KbPEI (408727, Sigma-Aldrich). For chemical priming, MSLN-CAR-NK-92 cells were collected, counted, and resuspended at a density of 5 × 10^5^ cells/mL in NK-92 complete culture medium supplemented with 200 U/mL recombinant human IL-2 (200-02-250UG, PeproTech). Cells were treated with 25KbPEI at a final concentration of 5 μg/mL for 12 h at 37 °C in a humidified incubator containing 5% CO_2_. Following treatment, cells were washed to remove residual 25KbPEI and resuspended in fresh medium. Cell viability and cell number were assessed before use in subsequent experiments.

### Cytotoxicity assay

NK cell-mediated cytotoxicity was evaluated using a CFSE/7-AAD–based flow cytometric assay. Target cancer cells were labeled with 1 µM CellTrace™ CFSE (C34554, Invitrogen) or CellTrace™ Far Red (C34564, Invitrogen) and incubated at 37 °C with 5% CO_2_ for 20 min. Labeled target cells were then co-cultured with effector NK-92, MSLN-CAR-NK-92, or CAR-Chem_NK-92 cells at the indicated effector-to-target (E:T) ratios for 4 h at 37 °C with 5% CO_2_. After co-culture, cells were stained with 7-Aminoactinomycin D (7-AAD; A1310, Invitrogen) for 20 min and subsequently fixed with 2% paraformaldehyde (PFA). The frequency of lysed target cells was determined using a CytoFLEX flow cytometer (Beckman Coulter) and analyzed with FlowJo software (Tree Star Inc.). Cytotoxicity was quantified as the percentage of 7-AAD^+^ cells among CFSE^+^ or CellTrace™ Far Red^+^ target cells.

### Degranulation assay

NK cell degranulation was assessed by measuring surface expression of CD107a (LAMP-1). Target cancer cells were labeled with 1 µM CellTrace™ Far Red and incubated at 37 °C with 5% CO_2_ for 20 min. NK-92, MSLN-CAR-NK-92, or CAR-Chem_NK-92 cells were co-cultured with target cells for 4 h at an E:T ratio of 1:1. Co-cultured cells were stained with anti-human CD107a (328646, BioLegend) for 30 min at room temperature. Degranulation was quantified as the percentage of CD107a^+^ cells within the Far Red^-^ NK cell population. Data were acquired using a CytoFLEX flow cytometer and analyzed with FlowJo software.

### Flow cytometry analysis

For surface marker staining, cells were washed with FACS buffer and incubated with fluorochrome-conjugated antibodies without permeabilization. For intracellular staining, cells were fixed in 2% paraformaldehyde (PFA) for 15 min and permeabilized with Foxp3 Perm Buffer for 20 min at room temperature in the dark. After permeabilization, cells were washed with FACS buffer and incubated with fluorochrome-conjugated antibodies. Flow cytometric data were acquired on a CytoFLEX flow cytometer and analyzed using FlowJo software. The following antibodies were used: APC anti-human Perforin Antibody (308111, BioLegend), BD Pharmingen™ PE Mouse Anti-Human IFN-γ (554701, BD Biosciences), PerCP anti-human CD69 Antibody (310927, BioLegend), BD Pharmingen™ PE Mouse anti-Human CD314 (NKG2D) (557940, BD Biosciences), PE anti-human CD335 (NKp46) Antibody (331907, BioLegend), PE anti-human CD197 (CCR7) Antibody (323204, BioLegend).

### Migration assay (transwell assay)

The migratory capacity of NK-92, MSLN-CAR-NK-92, and CAR-Chem_NK-92 cells was evaluated using a 24-well Transwell system with 8.0 µm pore size inserts (Falcon). A total of 3 × 10^5^ OVCAR3 cells were seeded into the lower chamber in 500 µL of serum-free medium and incubated for 12 h. NK-92, MSLN-CAR-NK-92, or CAR-Chem_NK-92 cells were labeled with 1 µM CellTrace™ CFSE and incubated at 37 °C with 5% CO_2_ for 20 min. A total of 5 × 10^5^ effector cells suspended in 100 µL of serum-free medium was then added to the upper Transwell inserts. After 24 h of incubation at 37 °C with 5% CO_2_, NK cells that had migrated to the lower chamber were collected and counted using a LUNA cell counter (Logos Biosystems). Migrated cells were also visualized using an EVOS fluorescence imaging system (Thermo Fisher Scientific).

### Time-lapse observation of NK cells

SKOV3 cells (2 × 10^4^) were labeled with 1 µM CellTrace™ Far Red and incubated at 37 °C in a humidified 5% CO_2_ incubator for 20 min prior to use in the assay. After labeling, the cells were washed and seeded in a culture plate and co-cultured with NK-92, MSLN-CAR-NK-92, or CAR-Chem_NK-92 cells at an effector-to-target (E:T) ratio of 2:1 in an Olympus FV3000 live-cell incubation imaging system. Time to NK–target attachment was defined as the time point at which the first NK cell established contact with an individual target cell. At each time point, the number of NK cells bound to target cells was divided by the total number of NK cells that established target contact during the observation period. Killing time was determined by real-time imaging and defined as the interval from initial NK binding to the time of target cell death. Overall killing activity was quantified as the fraction of dead target cells among total target cells within the field of view. Imaging was performed on a modified Olympus FV3000 confocal microscope equipped with a 20× objective lens, with focus maintained using an automated Z-drift compensation system. Acquired image data were analyzed using cellSens software (Olympus).

### *In vivo* animal experiments

Female BALB/c nude mice (5–6 weeks old) were purchased from JAbio (South Korea) and housed under the semi-SPF animal facility at CHA University (Seongnam, Korea). To establish subcutaneous xenografts, 1 × 10^7^ SKOV3 cells were resuspended in a 1:1 mixture of DPBS with Geltrex™ Flex LDEV-Free Reduced Growth Factor Basement Membrane Matrix (Thermo Fisher Scientific) and injected subcutaneously into the right flank of each mouse. Tumor growth was monitored using digital calipers, and tumor volume was calculated using the formula: tumor volume (mm^3^) = 0.5 × A × B^2^, where A represents the longest diameter and B represents the shortest diameter. Tumor implantation was defined as day 0. When tumors reached approximately 100 mm³, mice received a total of four intravenous injections of 5 × 10^6^ NK cells (NK-92, MSLN-CAR-NK-92, or CAR-Chem_NK-92) via the tail vein twice weekly on days 28, 31, 35, and 38. Tumor growth was monitored throughout the treatment period. The experimental endpoint was defined as the time at which tumors in the DPBS-treated control group reached approximately 1,000 mm³. At this endpoint, all mice were euthanized in a CO_2_ chamber using gradual-fill 100% CO_2_ at a displacement rate of 30% of the chamber volume per minute. Tumors were then excised, weighed, and processed for subsequent analysis. For biodistribution analysis, NK-92, MSLN-CAR-NK-92, or CAR-Chem_NK-92 cells were labeled with 1 µM CellTrace™ CFSE at 37 °C for 20 min and administered intravenously via the tail vein 24 h before euthanasia in the SKOV3 xenograft model. Mice were euthanized 24 h post-injection, and major organs, including the lung, heart, liver, spleen, kidney, and tumor, were harvested. Tissues were dissociated to generate single-cell suspensions. Analysis of CFSE-labeled NK cells was performed using a CytoFLEX flow cytometer (Beckman Coulter). CFSE-labeled NK cells were visualized by observing frozen tumor tissue (sectioned at 10 µm) under a Zeiss LSM510 microscope.

All animal experiments were approved by the Institutional Animal Care and Use Committee (IACUC 250123) and conducted in accordance with institutional guidelines.

### H&E staining

For histological evaluation, frozen tissue sections were fixed with 4% paraformaldehyde for 20 min at room temperature. After fixation, sections were washed with PBS and stained with hematoxylin for 3 min. The sections were then washed with PBS and rinsed in tap water for 3–10 min until appropriate nuclear staining was observed. After brief dehydration in 95% ethanol, sections were stained with eosin for 3 min and briefly rinsed with tap water. The sections were subsequently dehydrated in 95% ethanol for 1 min and briefly immersed in 100% ethanol, followed by clearing in xylene for 2–3 min. Finally, sections were mounted with Canada balsam, covered with coverslips, and dried overnight in a chemical fume hood.

### TUNEL assay

Apoptotic cell death in tumor tissues was assessed using the DeadEnd™ Fluorometric TUNEL System (G3250, Promega) according to the manufacturer’s instructions. Briefly, tumor tissues were embedded in OCT compound, cryosectioned at 10 μm thickness, and fixed with 4% paraformaldehyde for 15 min. After washing with PBS, tissue sections were permeabilized with Proteinase K buffer (20 µg/mL) for 15 min at room temperature. Sections were then equilibrated with equilibration buffer and incubated with the rTdT reaction mixture in a humidified chamber at 37 °C for 60 min in the dark. The reaction was terminated by incubation with 2× SSC, followed by PBS washing. After washing, nuclei were counterstained and mounted using DAPI mounting solution (Sigma-Aldrich, F6057). Fluorescence images were acquired using a Zeiss LSM510 microscope. TUNEL-positive cells were quantified as the percentage of TUNEL-positive nuclei among total DAPI-positive nuclei.

### Statistical analysis

All statistical analyses were performed using GraphPad Prism v9.3.0 (GraphPad Software, La Jolla, CA, USA), and the specific tests applied are indicated in the relevant figure legends. Data are presented as mean ± standard deviation (SD) or as box-and-whisker or violin plots, as specified in figure legends. Statistical significance was defined as ns, not significant; *p < 0.05, **p < 0.01, ***p < 0.001, and ****p < 0.0001.

## Results

### 25KbPEI-mediated chemical priming potentiates MSLN-CAR-NK-92 cytotoxicity and degranulation

**Figure 1:**
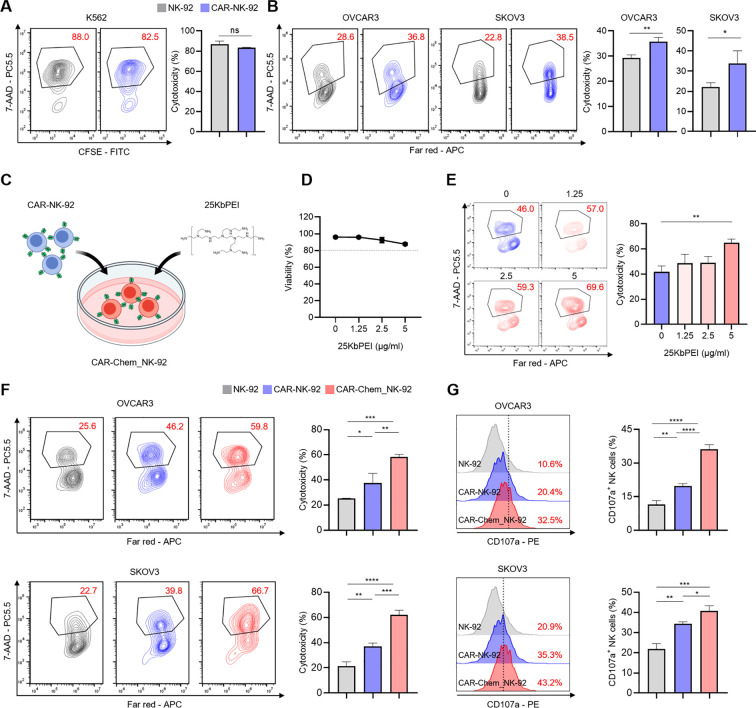
25KbPEI-mediated chemical priming enhances the cytotoxicity of MSLN-targeting CAR-NK-92. **(A)** Cytotoxic activity of parental NK-92 and CAR-NK-92 against K562 cells was assessed using a Far Red/7-AAD assay (E:T ratio = 2:1). **(B)** Antigen-specific cytotoxicity of NK-92 and CAR-NK-92 against MSLN-positive ovarian cancer cell lines (OVCAR3 and SKOV3) was evaluated using a Far Red/7-AAD assay (E:T ratio = 10:1). **(C)** Schematic illustration of the generation of CAR-Chem_NK-92 by treatment of CAR-NK-92 cells with 25KbPEI. **(D)** Cell viability of CAR-NK-92 following 25KbPEI treatment was determined at 12 h post-treatment using a trypan blue exclusion assay. **(E)** Dose-dependent effects of 25KbPEI on CAR-NK-92 cytotoxicity were evaluated against OVCAR3 cells using a Far Red/7-AAD assay (E:T ratio = 10:1). **(F)** Comparative cytotoxic activity of NK-92, CAR-NK-92, and CAR-Chem_NK-92 against OVCAR3 and SKOV3 cells (E:T ratio = 10:1). **(G)** Degranulation activity of NK-92, CAR-NK-92, and CAR-Chem_NK-92 following co-culture with OVCAR3 and SKOV3 cells was assessed by CD107a surface expression (E:T ratio = 1:1). All experiments were performed in three independent biological replicates. Statistical significance was determined using an unpaired Student**’**s t-test for panels A–B and one-way ANOVA followed by Tukey**’**s multiple comparisons test for panels E–G. Data are presented as mean ± SD. ns, not significant; *p < 0.05, **p < 0.01, ***p < 0.001, ****p < 0.0001.

To evaluate the impact of chemical priming on antigen-specific NK cell cytotoxicity, we utilized parental NK-92 cells and MSLN-CAR-expressing NK-92 cells (hereafter referred to as CAR-NK-92). The MSLN-CAR-NK-92 used in the present study were previously established and characterized (30). Consistent with preserved innate function, CAR-NK-92 exhibited cytotoxic activity comparable to parental NK-92 cells against the NK-sensitive leukemia target K562 (**Figure 1A**), indicating that CAR expression did not compromise baseline NK cell cytotoxicity. In contrast, CAR-NK-92 showed enhanced killing of MSLN-positive ovarian cancer cell lines, including OVCAR3 and SKOV3 (29), relative to parental NK-92 cells (**Figure 1B**), confirming antigen-specific activity mediated by the introduced CAR construct. To determine whether chemical priming further augments CAR-directed effector function, CAR-NK-92 were treated with 25KbPEI to generate chemically primed CAR-NK-92 (CAR-Chem_NK-92) (**Figure 1C**). 25KbPEI treatment induced a distinct morphological shift characterized by increased formation of colony-like aggregates (**Supplementary Figure 1**), consistent with chemical priming-associated phenotypic remodeling (21). Importantly, across the tested concentration range (0–5 μg/mL), 25KbPEI exposure did not affect NK cell viability (**Figure 1D**), indicating that the priming protocol was not cytotoxic. Functional analysis revealed that chemical priming enhanced CAR-NK-92 cytotoxicity against tumor targets in a dose-dependent manner, with maximal tumor cell killing observed at 5 μg/mL of 25KbPEI (**Figure 1E**). Based on these results, this concentration was selected for subsequent comparative analyses. Under optimized priming conditions, CAR-Chem_NK-92 exhibited significantly greater cytotoxicity than both parental NK-92 and CAR-NK-92 against OVCAR3 and SKOV3 targets (**Figure 1F**). This enhancement was accompanied by increased degranulation upon tumor engagement, as indicated by elevated CD107a surface expression (**Figure 1G**). Collectively, these findings demonstrate that chemical priming enhances both antigen-specific cytotoxicity and degranulation, establishing CAR-Chem_NK-92 as functionally reinforced effectors against ovarian cancer cells.

### CAR-Chem_NK-92 exhibits phenotypic and functional hallmarks of chemical priming

**Figure 2:**
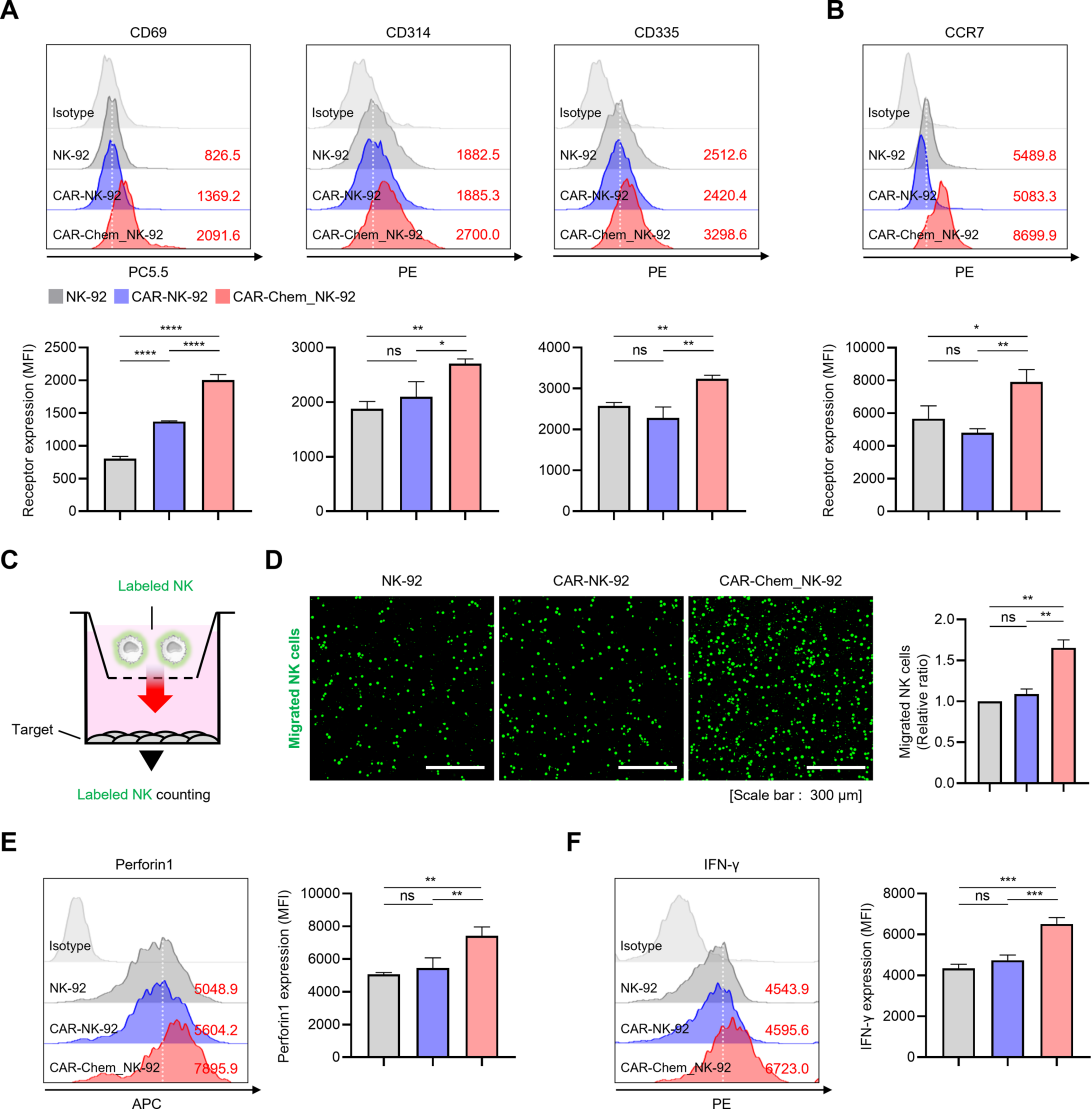
CAR-NK-92 acquires phenotypic and functional features of chemical priming following 25KbPEI treatment. **(A, B)** Flow cytometry analysis of activation and trafficking-associated receptors in NK-92, CAR-NK-92, and CAR-Chem_NK-92. Expression levels of CD69, CD314 (NKG2D), and CD335 (NKp46) **(A)**, as well as CCR7 **(B)**, are shown as representative histograms (top) and quantified as MFI (bottom). **(C)** Schematic of the Transwell-based migration assay used to evaluate NK cell migration toward target cells. **(D)** Representative fluorescence microscopy images (left) and quantification (right) of migrated NK cells. Data are presented as the relative number of migrated cells normalized to the NK-92 cells. Scale bar, 300 µm. **(E, F)** Flow cytometry analysis of effector molecule expression in NK-92, CAR-NK-92, and CAR-Chem_NK-92. Intracellular perforin expression **(E)** and IFN-γ expression **(F)** are shown as representative histograms (left) and quantified as MFI (right). All experiments were performed in three independent biological replicates. Statistical significance was determined using one-way ANOVA followed by Tukey**’**s multiple comparisons test. Data are shown as mean ± SD. ns, not significant; *p < 0.05, **p < 0.01, ***p < 0.001, ****p < 0.0001.

To determine whether chemical priming induces Chem_NK-like phenotypic remodeling in CAR-engineered NK cells, we analyzed activation markers, trafficking-associated receptors (21), and effector programs in CAR-Chem_NK-92. Flow cytometric analysis showed increased expression of activating receptors, including CD69, CD314 (NKG2D), and CD335 (NKp46), compared with non-primed CAR-NK-92 (**Figure 2A**), indicating enhanced activation readiness of CAR-Chem_NK-92. CAR-Chem_NK-92 also exhibited elevated expression of CCR7 (**Figure 2B**), suggesting acquisition of a trafficking-competent phenotype (24). Consistent with this, transwell migration assays demonstrated significantly enhanced tumor-directed migration compared with both CAR-NK-92 and NK-92 cells (**Figures 2C, D**). Chemical priming further reinforced the effector program, as evidenced by increased intracellular perforin accumulation and elevated IFN-γ expression (**Figures 2E, F**), indicating enhanced cytotoxic and cytokine-producing capacity.

### Chemical priming enhances CAR-NK-92 killing dynamics

**Figure 3:**
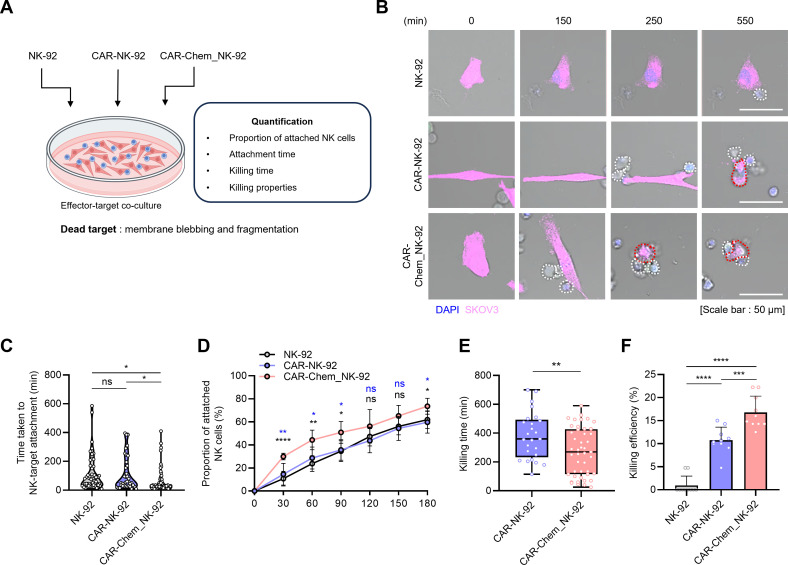
Live-cell imaging reveals enhanced target engagement and killing dynamics of CAR-Chem_NK-92. **(A)** Schematic of the live-cell imaging assay used to analyze NK cell-target interaction dynamics. Quantified parameters included time to initial NK cell-target attachment, proportion of NK cells engaged with targets, and killing time. Target cell death was defined by membrane blebbing and fragmentation. **(B)** Representative time-lapse images of Far Red-labeled SKOV3 cells co-cultured with NK-92, CAR-NK-92, or CAR-Chem_NK-92 at the indicated time points. Nuclei were stained with DAPI (blue). Dead target cells are indicated by red dashed lines, and NK cells are indicated by white dashed lines. Scale bar, 50 µm. **(C)** Time to first NK cell–target cell attachment was measured from the start of live-cell imaging to the first NK cell–target cell contact (n = 47 for NK-92, n = 38 for CAR-NK-92, and n = 67 for CAR-Chem_NK-92). **(D)** The proportion of target-bound NK cells over time was quantified at each time point during live-cell imaging analysis. (n = 9 for NK-92, n = 8 for CAR-NK-92, and n = 5 for CAR-Chem_NK-92). **(E)** Quantification of killing time, defined as the interval between initial NK cell-target contact and target cell death. (n = 25 for CAR-NK-92, and n = 45 for CAR-Chem_NK-92). **(F)** Overall killing efficiency, quantified as the percentage of dead target cells among total target cells within the imaging field. (n = 10 for NK-92, n = 10 for CAR-NK-92, and n = 10 for CAR-Chem_NK-92). Statistical significance was determined using an unpaired Student’s *t*-test for panel **(E)**, one-way ANOVA followed by Tukey’s multiple comparisons test for panels **(C, F)**, and two-way ANOVA followed by Tukey’s multiple comparisons test for panel **(D)** ns, not significant; *p < 0.05, **p < 0.01, ***p < 0.001, ****p < 0.0001.

To characterize the dynamic behavior of CAR-Chem_NK-92, we performed real-time live-cell imaging of NK–target interactions and quantified attachment and killing kinetics (**Figures 3A, B**). CAR-Chem_NK-92 exhibited a significantly shorter time to initial target engagement compared with CAR-NK-92 and NK-92 (**Figure 3C**), indicating more rapid target engagement. In addition, CAR-Chem_NK-92 cells showed a higher proportion of target-bound NK cells over time (**Figure 3D**), reflecting increased interaction frequency and/or stability. Importantly, chemical priming reduced the killing time, defined as the interval between initial contact and target cell death (**Figure 3E**), indicating accelerated cytotoxic execution. Consistent with these findings, overall killing efficiency was significantly increased in the CAR-Chem_NK-92 group (**Figure 3F**). Together, these results demonstrate that chemical priming enhances both the speed and efficiency of CAR-mediated tumor cell killing.

### Chemical priming enhances the *in vivo* antitumor efficacy and tumor infiltration of CAR-NK-92

**Figure 4:**
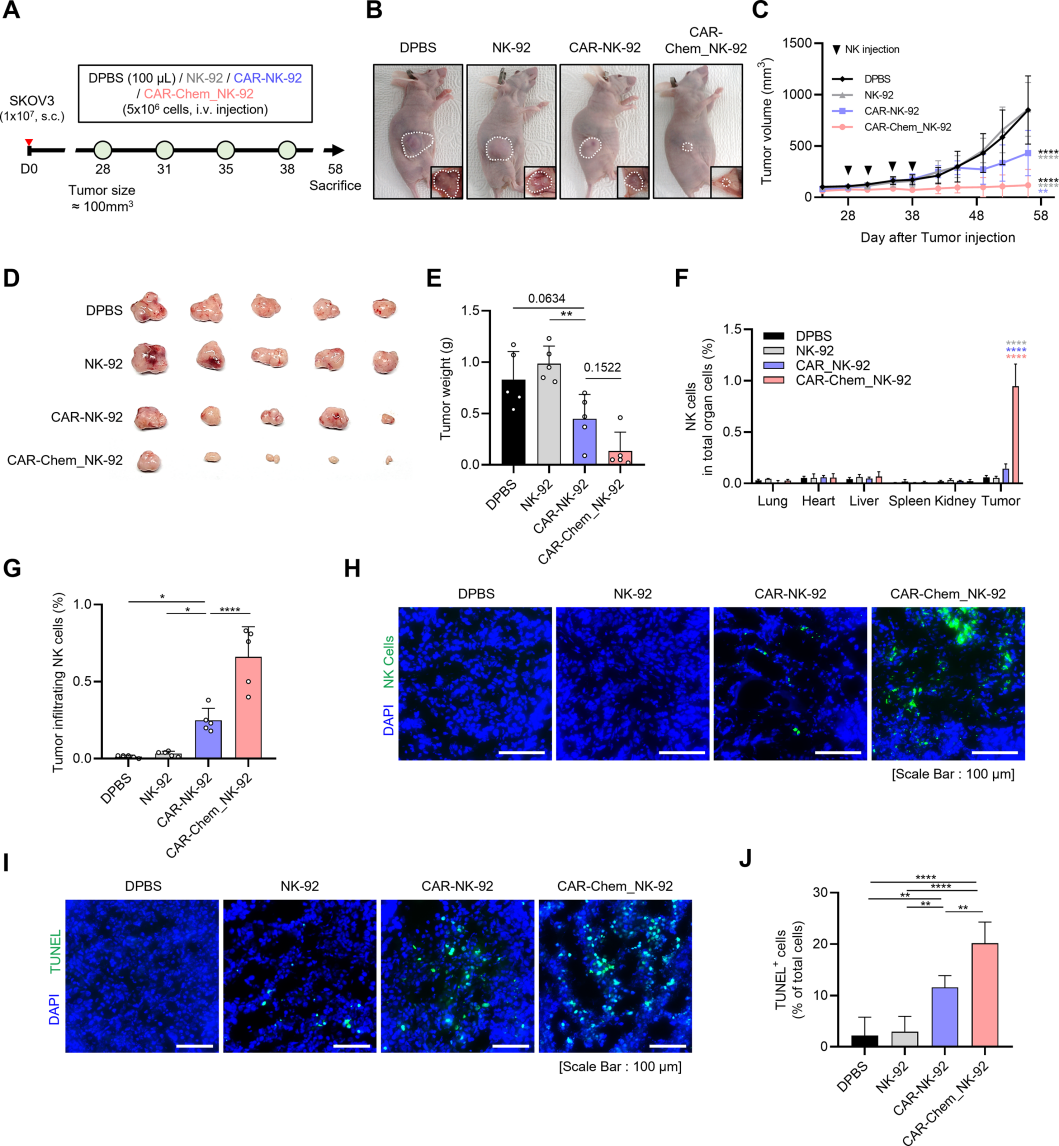
CAR-Chem_NK-92 displays enhanced *in vivo* antitumor activity. **(A)** Schematic of the *in vivo* experimental design. SKOV3 tumor-bearing BALB/c nude mice received repeated intravenous infusions of DPBS, NK-92, CAR-NK-92, or CAR-Chem_NK-92 according to the indicated schedule. **(B)** Representative images of tumor-bearing mice from each treatment group at the endpoint. Insets show magnified views of tumor regions. **(C)** Tumor growth curves of SKOV3 xenografts following treatment with DPBS, NK-92, CAR-NK-92, or CAR-Chem_NK-92. Arrows indicate the timing of NK cell administration. (each group n = 5). **(D)** Representative images of excised tumors from each treatment group at the endpoint. **(E)** Quantification of tumor weights from excised tumors at the experimental endpoint. (each group n = 5). **(F)** Distribution of NK cells across major organs, including the lung, heart, liver, spleen, kidney, and tumors, as determined by flow cytometry. (each group n = 3). **(G)** Flow cytometric quantification of tumor-infiltrating NK cells in each treatment group. (each group n = 5). **(H)** Representative fluorescence images of tumor sections showing infiltrating NK cells (green). Nuclei were stained with DAPI (blue). Scale bars, 100 μm. **(I)** Representative images of TUNEL staining in tumor tissue sections from each treatment group. Apoptotic cells are shown as TUNEL-positive signals (green), and nuclei were counterstained with DAPI (blue). Scale bars, 100 μm. **(J)** Quantification of TUNEL-positive cells in tumor sections. The frequency of apoptotic cells was calculated as the percentage of TUNEL-positive cells relative to total DAPI-positive nuclei. (each group n = 5). Statistical significance was assessed using one-way ANOVA followed by Tukey**’**s multiple comparisons test for panels **(E–G, J)**, and two-way ANOVA followed by Tukey**’**s multiple comparisons test for panel **(C)** ns, not significant; *p < 0.05, **p < 0.01, ****p < 0.0001.

To evaluate therapeutic efficacy *in vivo*, we established a SKOV3 subcutaneous xenograft model in BALB/c nude mice and initiated NK cell administration when tumors reached approximately 100 mm³, according to the indicated treatment schedule (**Figure 4A**). CAR-NK-92 treatment delayed tumor growth compared with DPBS and parental NK-92 controls, confirming antigen-specific antitumor activity. Notably, CAR-Chem_NK-92 exhibited the most pronounced tumor suppression throughout the treatment period (**Figures 4B, C**). Endpoint analysis revealed significantly reduced tumor burden in the CAR-Chem_NK-92 group, as demonstrated by decreased tumor size and weight compared with those in the control groups (**Figures 4D, E**). To assess trafficking *in vivo*, we analyzed NK cell distribution across tissues. CAR-Chem_NK-92 showed potential preferential accumulation within tumors without increased off-target organ distribution (**Figures 4F, G**). Immunofluorescence imaging further confirmed enhanced tumor infiltration of NK cells in the CAR-Chem_NK-92 group compared with CAR-NK-92 (**Figure 4H**). Importantly, treatment was well tolerated, as indicated by stable body weight throughout the treatment period (**Supplementary Figure 2**). Consistently, H&E staining of major organs, including the heart, lung, liver, kidney, and spleen, revealed no apparent histopathological abnormalities after NK cell administration (**Supplementary Figure 3**). Consistent with improved antitumor efficacy, TUNEL staining of tumor tissues revealed increased apoptosis in tumor tissues from CAR-Chem_NK-92-treated mice (**Figures 4I, J**). Collectively, these findings demonstrate that chemical priming significantly enhances the *in vivo* antitumor efficacy of CAR-NK-92 by improving tumor infiltration and cytotoxic function.

## Discussion

Adoptive NK cell therapy using CAR-engineered NK cells represents a promising paradigm in cancer immunotherapy, offering a safer, off-the-shelf alternative to T cell-based therapies (31). However, the clinical efficacy of CAR-NK cells against solid tumors remains limited, largely due to inefficient tumor infiltration and impaired effector function within the TME (11). In this study, we demonstrate that transient chemical priming with 25KbPEI enhances multiple functional properties of MSLN-targeting CAR-NK-92 cells, including tumor-directed migration, cytotoxic effector activity, and tumor cell killing dynamics.

Insufficient homing of NK cells to tumor sites is a major barrier to effective NK cell-based therapies in solid tumors (32). Here, we show that chemical priming upregulates trafficking-associated receptors, including CCR7, and significantly enhances tumor-directed migration *in vitro*. Importantly, this phenotype was also observed *in vivo*, where CAR-Chem_NK-92 exhibited increased tumor infiltration without apparent accumulation in non-target organs. These findings suggest that chemical priming may improve tumor localization of CAR-NK cells. However, the present study was not designed to distinguish whether the enhanced tumor accumulation resulted from increased chemotaxis, enhanced adhesion, improved tissue retention, or increased survival after tumor infiltration. Further studies using defined chemokine gradients, chemokine receptor blockade, and real-time trafficking analysis will be necessary to clarify the mechanisms underlying the improved tumor homing phenotype.

In addition to enhanced trafficking, chemical priming reinforced several cytotoxic features of CAR-NK-92, including perforin accumulation, degranulation, and accelerated tumor-cell killing kinetics. Live-cell imaging analyses consistently demonstrated more rapid target engagement and shortened binding-to-death intervals in CAR-Chem_NK-92.

The enhanced functional capacity of CAR-Chem_NK-92 may be related to metabolic changes induced by 25KbPEI exposure. In our previous studies, chemical priming was associated with rapid Ca²^+^ influx and enhanced mitochondrial oxidative phosphorylation (21, 22). Such metabolic reinforcement may support CAR-NK-92 effector functions under metabolically stressful TME conditions. However, the present study did not directly investigate whether Ca²^+^ signaling or metabolic reprogramming causally mediates the enhanced trafficking, cytotoxicity, or killing dynamics observed in CAR-Chem_NK-92. Therefore, these pathways should be considered potential contributing mechanisms rather than definitive causal drivers. Further mechanistic studies incorporating metabolic profiling, Ca²^+^ influx analyses, and pathway-specific perturbation experiments will be required to define the biological basis of chemical priming in CAR-NK cells.

Cytokine-based priming, particularly with IL-12, IL-15, and IL-18, is a well-established strategy for enhancing NK cell function through induction of memory-like NK phenotypes, increased cytokine responsiveness, and improved persistence (19, 20). In contrast, 25KbPEI-mediated chemical priming may provide a mechanistically distinct and potentially complementary approach rather than a replacement for cytokine priming. Chemical priming is a transient, non-genetic, and modular intervention that can be incorporated into existing CAR-NK manufacturing workflows without modification of the CAR construct itself. In the present study, chemical priming preferentially enhanced functional properties particularly relevant to solid tumor therapy, including tumor-directed migration, cytotoxic payload accumulation, degranulation, and rapid tumor cell killing kinetics. These findings suggest that chemical priming may preferentially reinforce immediate effector readiness and trafficking competence, thereby addressing key bottlenecks limiting CAR-NK efficacy in solid tumors, such as inefficient tumor infiltration and delayed cytotoxic execution. Nevertheless, direct comparative studies will be necessary to determine whether chemical priming provides distinct advantages over cytokine priming or whether the two approaches may function synergistically.

Despite these promising results, several limitations of this study should be acknowledged. First, this study primarily utilized NK-92-derived CAR-NK cells. Although NK-92 cells provide a robust and scalable experimental platform, they are immortalized cells and may not fully recapitulate the biological properties of primary human NK cells or iPSC-derived NK cells. Therefore, validation in clinically relevant CAR-NK platforms will be essential. Second, although CCR7 upregulation correlated with the enhanced migration of CAR-Chem_NK-92, the precise chemokine signaling pathways responsible for improved tumor homing remain to be fully elucidated. Ovarian cancer cells can produce diverse chemokines, including CXCL1/2/3, CXCL8, and CCL20 (33), while NK cell trafficking is regulated by multiple receptors, including CXCR1/3/4/6, and CCR7 (32). In the present study, CAR-Chem_NK-92 showed increased CCR7 expression, and our previous Chem_NK study also demonstrated upregulation of additional chemokine receptors, such as CCR5 and CXCR1, following chemical priming (21). Therefore, the enhanced migration of CAR-Chem_NK-92 may reflect broader responsiveness to tumor-derived chemotactic cues rather than activation of a single CCR7-dependent pathway. Third, the current study employed an immunodeficient xenograft model, which is useful for evaluating human tumor growth and CAR-NK cell activity *in vivo*, but does not fully capture the complexity of endogenous immune responses or the broader immunosuppressive TME. Future studies using immunocompetent or humanized tumor models will therefore be important to further evaluate the immunomodulatory effects and translational relevance of chemically primed CAR-NK cells.

In conclusion, our findings suggest that 25KbPEI-mediated chemical priming enhances the antitumor activity of MSLN-CAR-NK-92 in a preclinical solid tumor model by improving tumor localization and cytotoxic killing dynamics. While these results support chemical priming as a potentially useful non-genetic strategy for enhancing selected CAR-NK cell functions, further validation using primary or iPSC-derived CAR-NK cells, immunocompetent or humanized tumor models, and direct mechanistic analyses will be necessary to define its translational relevance.

## Data Availability

The raw data supporting the conclusions of this article will be made available by the authors, without undue reservation.
