# Supplementary material for: Chemical priming potentiates mesothelin-targeting chimeric antigen receptor-engineered NK-92 antitumor activity by improving tumor trafficking and cytotoxic killing dynamics
Source: Front Immunol. 2026 Jun 1;17:1860442. doi: 10.3389/fimmu.2026.1860442 (PMC13265450; doi:10.3389/fimmu.2026.1860442)
Supplement: Supplementary file 1 [file Table1.docx]

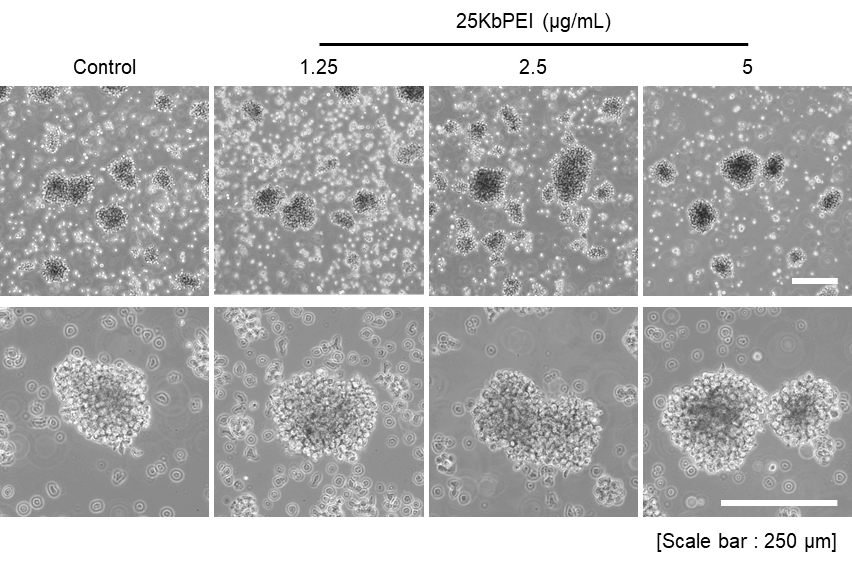


Supplementary Figure 1. 25KbPEI induces concentration-dependent morphological changes in NK cells.

Representative bright-field images of NK cells following treatment with increasing concentration of 25KbPEI (0–5 μg/mL), showing the formation of cell aggregates in a dose-dependent manner. Images are shown at low magnification (top) and high magnification (bottom). Scale bars, 250 μm.

Supplementary Figure 2. CAR-Chem_NK-92 treatment does not affect body weight in tumor-bearing mice.

Body weight of SKOV3 tumor-bearing mice treated with DPBS, NK-92, CAR-NK-92, or CAR-Chem_NK-92 was monitored throughout the experimental period. (each group n = 5).


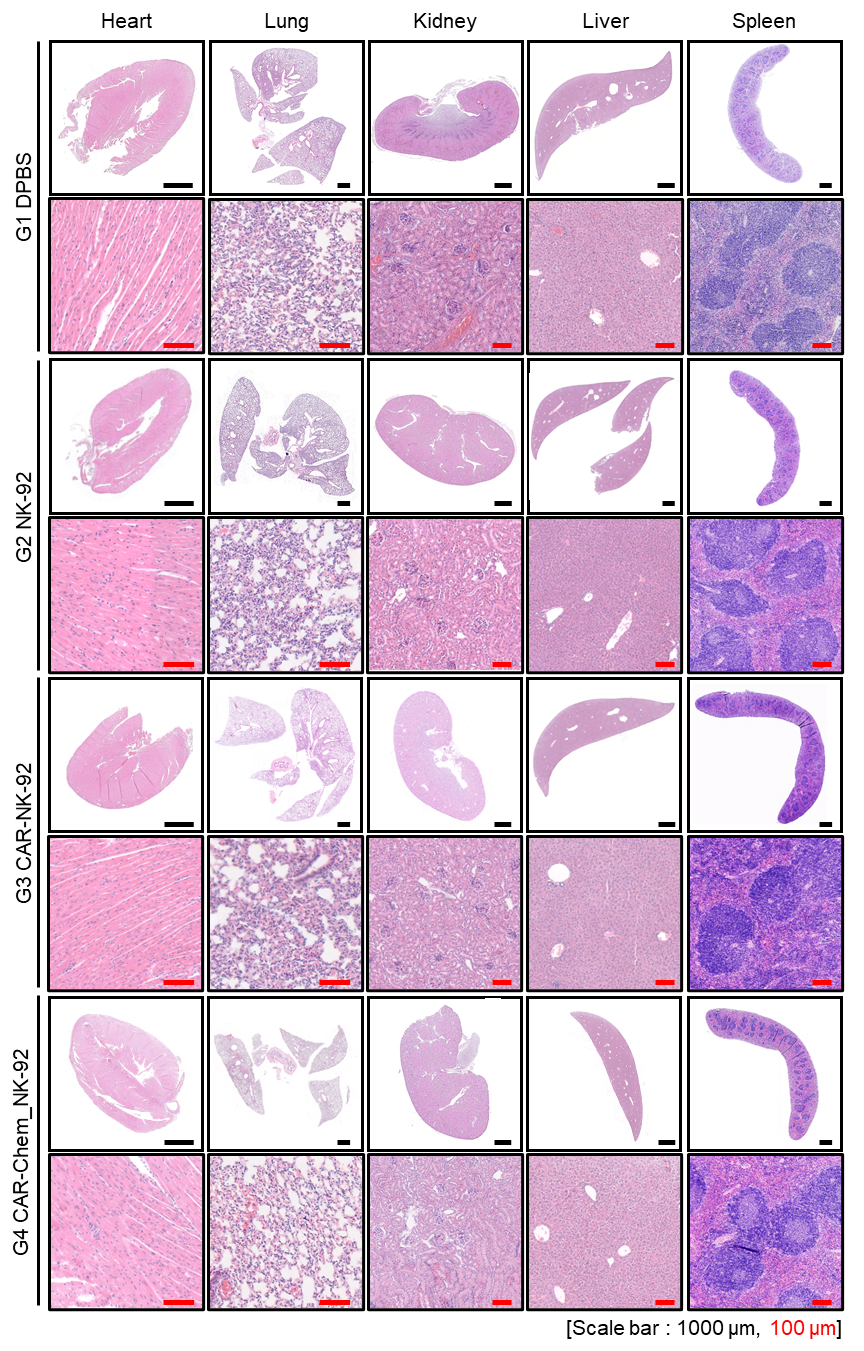


Supplementary Figure 3. CAR-Chem_NK-92 treatment does not induce apparent histopathological abnormalities in major organs.

Representative hematoxylin and eosin (H&E) stained images of the heart, lung, kidney, liver, and spleen from each treatment group for histopathological toxicity evaluation. Black scale bars, 1000 μm, red scale bars, 100 μm.
